# Complexity in the genetic architecture of leukoaraiosis in hypertensive sibships from the GENOA Study

**DOI:** 10.1186/1755-8794-2-16

**Published:** 2009-04-07

**Authors:** Jennifer A Smith, Stephen T Turner, Yan V Sun, Myriam Fornage, Reagan J Kelly, Thomas H Mosley, Clifford R Jack, Iftikhar J Kullo, Sharon LR Kardia

**Affiliations:** 1 Department of Epidemiology, School of Public Health, University of Michigan, Ann Arbor, MI, USA; 2 Division of Nephrology and Hypertension, Department of Internal Medicine, Mayo Clinic and Foundation, Rochester, MN, USA; 3 Human Genetics Center and Institute of Molecular Medicine, University of Texas-Houston Health Science Center, Houston, TX, USA; 4 Department of Medicine, University of Mississippi Medical Center, Jackson, MS, USA; 5 Department of Diagnostic Radiology, Mayo Clinic and Foundation, Rochester, MN, USA; 6 Division of Cardiovascular Diseases, Department of Internal Medicine, Mayo Clinic and Foundation, Rochester, MN, USA

## Abstract

**Background:**

Subcortical white matter hyperintensity on magnetic resonance imaging (MRI) of the brain, referred to as leukoaraiosis, is associated with increased risk of stroke and dementia. Hypertension may contribute to leukoaraiosis by accelerating the process of arteriosclerosis involving penetrating small arteries and arterioles in the brain. Leukoaraiosis volume is highly heritable but shows significant inter-individual variability that is not predicted well by any clinical covariates (except for age) or by single SNPs.

**Methods:**

As part of the Genetics of Microangiopathic Brain Injury (GMBI) Study, 777 individuals (74% hypertensive) underwent brain MRI and were genotyped for 1649 SNPs from genes known or hypothesized to be involved in arteriosclerosis and related pathways. We examined SNP main effects, epistatic (gene-gene) interactions, and context-dependent (gene-environment) interactions between these SNPs and covariates (including conventional and novel risk factors for arteriosclerosis) for association with leukoaraiosis volume. Three methods were used to reduce the chance of false positive associations: 1) false discovery rate (FDR) adjustment for multiple testing, 2) an internal replication design, and 3) a ten-iteration four-fold cross-validation scheme.

**Results:**

Four SNP main effects (in *F3*, *KITLG*, *CAPN10*, and *MMP2*), 12 SNP-covariate interactions (including interactions between *KITLG *and homocysteine, and between *TGFB3 *and both physical activity and C-reactive protein), and 173 SNP-SNP interactions were significant, replicated, and cross-validated. While a model containing the top single SNPs with main effects predicted only 3.72% of variation in leukoaraiosis in independent test samples, a multiple variable model that included the four most highly predictive SNP-SNP and SNP-covariate interactions predicted 11.83%.

**Conclusion:**

These results indicate that the genetic architecture of leukoaraiosis is complex, yet predictive, when the contributions of SNP main effects are considered in combination with effects of SNP interactions with other genes and covariates.

## Background

Stroke and dementia are age-related neurological disorders that cause considerable morbidity and financial burden in the US, with a lifetime risk for developing one or both of these disorders greater than 1 in 3 [1]. Risk factors for stroke and dementia overlap in part with those for cardiovascular disease (including age, sex, tobacco use, hypertension, diabetes mellitus, and low physical activity), but it has been established that both disorders have a significant genetic component that operates independently of these risk factors [2]. Although several rare genetic variations have been identified that are associated with significantly elevated risk of stroke or dementia, the vast majority of genes that influence risk for these disorders remain unidentified.

In an effort to increase the statistical power for detecting genetic variants that have small effects on the development of late-life endpoints, such as stroke and dementia, quantitative endophenotypes are often used as an indicator for risk of future disease. Endophenotypes may be closer in the biological hierarchy to the underlying genetic processes, including the influence of gene-environment interactions, and thus may have a larger genetic component than clinical disease endpoints.

Ischemic damage to the subcortical white matter that manifests as white matter hyperintensity on magnetic resonance imaging (MRI) of the brain, referred to as leukoaraiosis, is associated with increased risk of stroke and dementia [3-5]. One of the strongest predictors of leukoaraiosis is elevated blood pressure [6], in particular, inadequate blood pressure control in persons with hypertension [7]. Hypertension is thought to contribute to the pathology of leukoaraiosis through accelerating the age-related process of arteriosclerosis resulting in ischemic damage to penetrating small arteries and arterioles in the subcortical white matter of the brain [8]. This connection between hypertension and leukoaraiosis motivated the measurement of this subclinical phenotype in a subsample of the Genetic Epidemiology Network of Arteriopathy (GENOA) study of hypertensive sibships [9].

In the GENOA cohort, the heritability of the logarithm-transformed measure of leukoaraiosis volume was 0.80, which decreased to 0.68 after adjustment for sex, age, systolic blood pressure, and brain volume [10]. In other studies, the heritability of white matter hyperintensities on MRI was estimated to be 0.73 in a study of male twins [11] and 0.55 in the Framingham Heart Study [12].

To begin to explore the genetic architecture of this trait, we identified SNPs (single nucleotide polymorphisms) in 268 genes that have been previously identified as playing a role in processes related to arteriosclerosis including blood pressure regulation, vascular wall biology, oxidative stress, inflammation, obesity, diabetes, and lipoprotein metabolism. The goal of the present study was to investigate the contributions, covariation, and interaction among the many hypothesized genetic and environmental factors that may influence inter-individual variation in leukoaraiosis. Using a systematic approach that simultaneously investigates the contributions of these factors (as main effects or as part of interactions) and their underlying covariation, this study is a first step toward understanding the complexity of the genetic architecture of leukoaraiosis in order to begin to build multivariable models that can reliably predict levels of structural brain injury that may result from a person's unique combination of risk factors.

## Methods

### Study Population

The 777 study participants consisted of non-Hispanic white adults (322 male and 455 female) from 357 sibships that were initially enrolled in the Genetic Epidemiology Network of Arteriopathy (GENOA) study, a community-based study of hypertensive sibships that aims to identify genes influencing blood pressure (BP) [9,13]. The study was approved by the Institutional Review Board of Mayo Clinic, Rochester MN, and written informed consent was obtained from each participant. In the initial phase of the GENOA study (9/1995 to 6/2001), sibships containing ≥ 2 individuals with essential hypertension diagnosed before age 60 years were selected for participation. Participants returned for a second phase of the study (12/2000 to 6/2004) which included a physical examination and measurement of conventional and novel risk factors.

As an ancillary study of GENOA conducted between August 2001 and May 2006, the Genetics of Microangiopathic Brain Injury (GMBI) study was undertaken to determine susceptibility genes for ischemic brain injury. Leukoaraiosis was quantified by magnetic resonance imaging (MRI) in 916 non-Hispanic white subjects who participated in the second phase of the GENOA study, had a sibling willing and eligible to participate in the GMBI study, and had no history of stroke or neurological disease and no implanted metal devices. The median time between the second GENOA examination and the GMBI brain MRI was 11.9 months. Brain MRIs were suitable for analysis in 883 of the 916 participants; in the 33 without analyzable data, the most common reasons were unsuspected prior brain infarctions, masses, metallic artifacts, and failure to complete the MRI. After removing individuals who did not have genotyping data available, the final analysis subset consisted of 777 GMBI participants.

### Clinical Assessments and Covariate Definitions

The diagnosis of hypertension was established based on BP levels measured at the study visit (>140 mmHg average systolic BP or >90 mmHg average diastolic BP) or a prior diagnosis of hypertension and current treatment with antihypertensive medications. Height was measured by stadiometer, weight by electronic balance, and body mass index (BMI) was calculated as weight in kilograms divided by the square of height in meters. Resting systolic blood pressure (SBP) and diastolic blood pressure (DBP) were measured by a random zero sphygmomanometer, and pulse pressure was calculated as the difference between SBP and DBP. A person was considered having ever smoked if they had smoked more than 100 cigarettes in their lifetime, was considered to have coronary heart disease if they had ever experienced a myocardial infarction or had surgery for a blocked artery in the heart or neck (carotid artery), and was considered obese if they had a BMI > 30 kg/m^2^.

Blood was drawn by venipuncture after an overnight fast. Serum triglycerides (TG), creatinine, total cholesterol, and high-density lipoprotein (HDL) cholesterol were measured by standard enzymatic methods on a Hitachi 911 Chemistry Analyzer (Roche Diagnostics, Indianapolis IN), and low-density lipoprotein (LDL) cholesterol levels were calculated using the Friedewald formula [14]. Five novel vascular risk factors including C-reactive protein, homocysteine, fibrinogen, Lp(a), and LDL particle size were also measured. C-reactive protein was measured by a highly sensitive immunoturbidimetric assay [15], fibrinogen was measured by the Clauss (clotting time based) method [16], and plasma homocysteine was measured by high-pressure liquid chromatography. Lp(a) in serum was measured by an immunoturbidimetric assay using the SPQ™ Test System (Diasorin, Stillwater MN) as previously described [17], and LDL particle size was measured by polyacrylamide gel electrophoresis [18]. Level of physical activity was calculated as a continuous variable based on the self-reported average number of hours per day that the subject engaged in heavy, moderate, and sedentary activities according the following formula: 2*Heavy + Moderate – 2*Sedentary.

Leukoaraiosis volume (cm^3^) was obtained via magnetic resonance imaging (MRI) in a separate clinical visit. All MRI scans were performed on identically equipped Signa 1.5 T MRI scanners (GE Medical Systems, Waukesha, WI, USA) and images were centrally processed at the Mayo Clinic. Symmetric head positioning with respect to orthogonal axes was verified by a series of short scout scans. Total intracranial volume (head size) was measured from T1-weighted spin echo sagittal images, each set consisting of 32 contiguous 5 mm thick slices with no interslice gap, field of view = 24 cm, matrix = 256 × 192, obtained with the following sequence: scan time = 2.5 min, echo time = 14 ms, repetitions = 2, replication time = 500 ms [19]. Total brain and leukoaraiosis volumes were determined from axial fluid-attenuated inversion recovery (FLAIR) images, each set consisting of 48 contiguous 3-mm interleaved slices with no interslice gap, field of view = 22 cm, matrix = 256 × 160, obtained with the following sequence: scan time = 9 min, echo time = 144.8 ms, inversion time = 2,600 ms, repetition time = 26,002 ms, bandwidth = +/- 15.6 kHz, one signal average. A FLAIR image is a T2-weighted image with the signal of the cerebrospinal fluid nulled, such that brain pathology appears as the brightest intracranial tissue. Interactive imaging processing steps were performed by a research associate who had no knowledge of the subjects' personal or medical histories or biological relationships. A fully automated algorithm was used to segment each slice of the edited multi-slice FLAIR sequence into voxels assigned to one of three categories: brain, cerebrospinal fluid, or leukoaraiosis. The mean absolute error of this method is 1.4% for brain volume and 6.6% for leukoaraiosis volume, and the mean test-retest coefficient of variation is 0.3% for brain volume and 1.4% for leukoaraiosis volume [20]. White matter hyperintensities in the corona-radiata and periventricular zone, as well as central gray infarcts (ie, lacunes) were included in the global leukoaraiosis measurements. Brain scans with cortical infarctions were excluded from the analyses because of the distortion of the leukoaraiosis volume estimates that would be introduced in the automated segmentation algorithm.

### Genotyping

One thousand nine hundred and fifty six SNPs from 268 genes known or hypothesized to be involved in blood pressure regulation, lipoprotein metabolism, inflammation, oxidative stress, vascular wall biology, obesity and diabetes were identified from the genetic association literature and positional candidate gene studies [21]. SNPs were chosen based on a number of different criteria including the published literature, non-synonymous SNPs with a minor allele frequency (MAF) > 0.02, and tag SNPs identified using public databases such as dbSNP  and the Seattle SNPs database .

DNA was isolated using the PureGene DNA Isolation Kit from Gentra Systems (Minneapolis MN). Genotyping, based on polymerase chain reaction (PCR) amplification techniques, was conducted at the University of Texas-Health Sciences Center at Houston using the TaqMan assay and ABI Prism^® ^Sequence Detection System (Applied Biosystems, Foster City CA). Primers and probes are available from the authors upon request. Quality control measures for genotyping assays included robotic liquid handling, separate pre- and post-PCR areas, standard protocols and quality control analyses including 5% duplicates, positive and negative controls, computerized sample tracking, and data validity checks. After removal of SNPs that were monomorphic in the study sample, 1649 SNPs remained for analysis (see Additional file 1).

### Statistical Analysis

All analyses were carried out using the R statistical language, version 2.8 [22]. Covariate correlations were estimated using Pearson's product moment correlation. Linkage disequilibrium (LD), as measured by r^2 ^[23], was estimated using an expectation maximization (EM) algorithm. Hardy-Weinberg Equilibrium was assessed using a chi-square test or Fisher's exact test if a genotype class had less than 5 individuals [24]. Variables that showed a large deviation from a normal distribution in diagnostic plots, including leukoaraiosis, were transformed by taking the natural logarithm. The outcome variable for all models is the residual value of the natural logarithm of leukoaraiosis volume (cm^3^) after adjustment for age, sex, and total brain volume.

In the first stage of the analysis, we tested for association between leukoaraiosis and each of the predictor variables (SNPs and quantitative covariates) using least-squares linear regression methods [24,25]. Categorical covariates were modeled using logistic regression [25]. We also tested for association between each SNP and covariate to identify potential confounders. To determine whether interactions among predictors explained additional variation in the outcome, we tested pairwise interactions among all possible pairs of predictors (i.e. SNP-SNP, SNP-covariate, and covariate-covariate interactions) for all covariates and the 444 SNPs that had a model p-value < 0.2 in the association testing described above. Associations involving interactions were assessed with a partial F test, which compares a full model that includes both the interaction terms and the main effects of the variables comprising the interaction terms to a reduced model that includes only the main effects. Models with a p-value < 0.1 (for single variable models) or a partial F p-value < 0.1 (for models with interaction terms) were evaluated in the next stage of analysis.

To reduce false positives we used three different approaches: 1) adjustment for multiple testing using the False Discovery Rate (FDR) < 0.30 [26], 2) internal replication with two subsets of the data (constructed so individuals were unrelated within subset), and 3) four-fold cross-validation (repeated 10 times) [27]. To create internal replication subsets, we randomly selected one sibling from each sibship without replacement to create subset 1 and then randomly selected another sibling from each sibship to create subset 2. The GMBI cohort contained a small number of singletons (ie, subjects who had no enrolled sibling) that were equally divided between the two samples. Associations that had a p-value < 0.1 in both subsets were considered internally replicated if the effect of the genotype was homogeneous among subsets (the partial F p-value > 0.05 from a test of the interaction between subset designation and the predictors(s) under consideration).

Cross-validation significantly reduces false positive results by eliminating associations that lack predictive ability in independent test samples. For each association, we performed four-fold cross-validation by dividing the full sample into four equally sized groups. Three of the four groups were combined into a training dataset, and the modeling strategy outlined above was carried out to estimate model coefficients. These coefficients were then applied to the fourth group, the testing dataset, to predict the value of the outcome variable for each individual in this independent test sample. This process was repeated for each of the four testing sets. Predicted values for all individuals in the test set were then subtracted from their observed values, yielding the total residual variability (SSE), . The total variability in the outcome (SST) – the difference between each individual's observed value and the mean value for the outcome – was then calculated, . In order to estimate the proportion of variation in the outcome predicted in the independent test samples, the cross-validated R^2 ^(CV R^2^) was calculated as follows: . This cross-validation method provides a more accurate measure of the predictive ability of the genetic models and will be negative when the model's predictive ability is poor. Because random variations in the sampling of the four mutually exclusive test groups can potentially impact the estimates of CV R^2^, this procedure was repeated 10 times and the CV R^2 ^values were averaged [27].

Univariate associations were considered cross-validated if the average percent variation predicted in independent test samples was greater than 0.5% and interactions were considered cross-validated if the difference in average percent variation predicted in independent test samples between the full model containing the interaction term and the reduced model containing only main effect terms was greater than 0.5%. Using permutation testing on the models investigated in this paper, we found that the probability of observing a CV R^2 ^× 100 greater than 0.5% by chance alone was less than 5%. That is, Pr(CV R^2 ^× 100 > 0.5%) < 0.05 under the null hypothesis of no association. Due to small cell sizes (<4 subjects in a particular class), 0.3% of the SNP-covariate interaction models and 2.3% of the SNP-SNP interaction models were unable to complete the cross-validation procedure.

All single SNP or interaction models that passed the three different approaches for reducing false positives (FDR, internal replication, and cross-validation) were modeled using linear mixed effects (LME) [28], which accounts for the sibship structure among GMBI study participants while retaining a valid type I error rate [29]. Associations with a p-value <0.1 in the F test (described above) but a p-value >0.1 from the likelihood ratio test of the appropriate full and reduced mixed effects models were considered to be associations due to family structure and were removed from the results.

To visualize the genetic architecture of leukoaraiosis volume, we applied a novel data visualization scheme, the KGraph, described in Kelly et al. [30]. The KGraph was developed for the visualization of genetic association results and the underlying relationships among predictors such as SNP-SNP frequency correlations (i.e. LD), SNP-covariate associations, and covariate-covariate correlations. It simultaneously displays both significant univariate associations and pairwise interactions with the outcome of interest, leukoaraiosis volume, as well as the underlying correlation structure among the predictor variables.

In the final step, multivariable linear regression models combining the most predictive SNPs, covariates, and their interactions were constructed. The top four single SNP, SNP-covariate, and SNP-SNP interaction models were chosen for multiple variable modeling based on the following criteria: 1) passed all three filters to reduce false positive associations (FDR, internal replication, and cross-validation), 2) had the highest CV R^2 ^values of the particular modeling strategy, and 3) didn't involve SNPs in strong LD with SNPs already included in the multiple variable model. Percent variation in leukoaraiosis volume explained by each model was assessed with the adjusted R^2 ^value, and predictive ability of the models was assessed by four-fold, ten-iteration cross-validation (CV R^2 ^value).

## Results

### Descriptive Statistics

**Figure 1:**
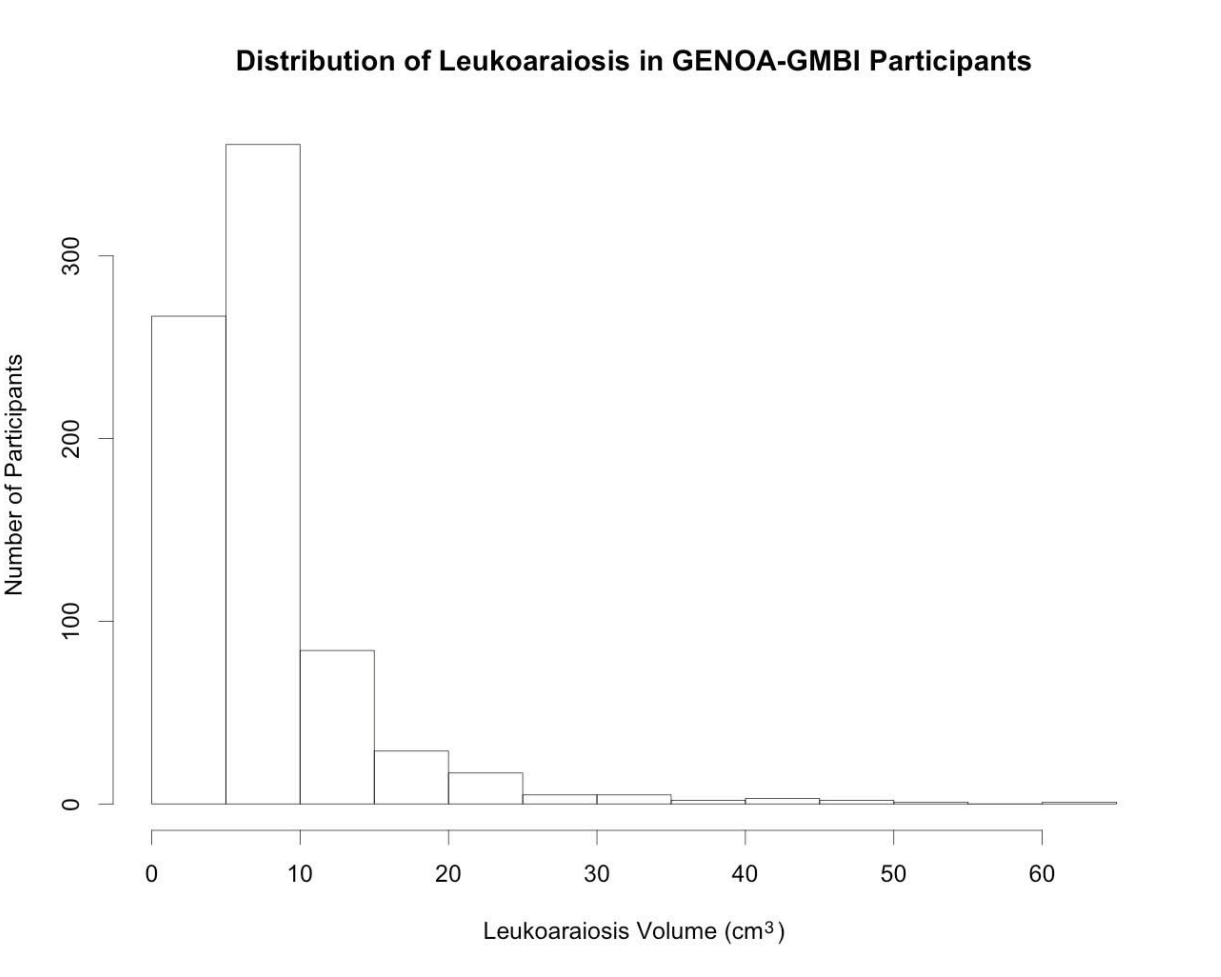
**The Distribution of Leukoaraiosis Volume (cm^3^) in GENOA-GMBI Study Participants**.

Descriptive statistics of the clinical covariates and outcomes are shown in Table 1. The mean age of the participants was 59.7 years and 58.6% of participants were female. Participants had a mean BMI of 30.5 kg/m^2^, waist-to-hip ratio of 0.91, SBP of 131.4 mmHg, and DBP of 74.0 mmHg. The distribution of leukoaraiosis is shown in Figure 1. The mean volume of leukoaraiosis was 7.80 cm^3 ^(median = 5.92 cm^3^) and the mean brain size was 1159 cm^3^. Allele and genotype frequencies, rs numbers from dbSNP, SNP positions and annotations (synonymous, non-synonymous, intron, etc), and test results for Hardy-Weinberg equilibrium are reported in Additional file 1.

**Table 1:** Descriptive Statistics for Study Participants

	**Full Sample**		**Subset 1**		**Subset 2**		**Association with Outcome^a^ in Full Sample**
	**N**	**Mean (± SD)**	**N**	**Mean (± SD)**	**N**	**Mean (± SD)**	**β estimate**
Leukoaraiosis volume (cm^3^)	777	7.80 (6.31)	316	7.90 (6.18)	316	7.95 (6.49)	NA
Age, years^b^	777	59.7 (10.1)	316	59.9 (10.4)	316	59.9 (9.7)	0.0295***
BMI, kg/m^2^	777	30.5 (5.8)	316	30.3 (5.8)	316	30.8 (5.7)	-0.003
Height, cm	777	168 (9.2)	316	169 (9.6)	316	169 (9.4)	-0.0003
Weight, kg	777	86.3 (18.6)	316	86.9 (18.8)	316	87.9 (18.5)	-0.0009
Waist-to-hip ratio	777	0.91 (0.11)	316	0.92 (0.12)	316	0.92 (0.10)	0.2162
SBP, mm Hg	776	131.4 (16.5)	316	131.8 (17.0)	315	131.6 (16.3)	0.0015
DBP, mm Hg	776	74.0 (9.0)	316	73.9 (8.6)	315	74.5 (9.1)	0.0052**
Pulse pressure, mm Hg	776	57.4 (15.3)	316	57.9 (15.8)	315	57.1 (15.0)	8.1 E-06
Total cholesterol, mg/dL	777	197.4 (33.7)	316	193.4 (33.4)	316	197.4 (32.3)	-0.0008
Triglycerides (log), mg/dL	777	4.93 (0.52)	316	4.92 (0.54)	316	4.99 (0.48)	0.0354
HDL cholesterol, mg/dL	777	51.9 (14.6)	316	51.4 (14.8)	316	50.0 (13.6)	0.0007
LDL cholesterol, mg/dL	777	120.1 (31.9)	316	116.9 (31.7)	316	121.1 (31.5)	-0.0011*
LDL particle size, Å	777	270.2 (5.0)	316	270.3 (5.1)	316	269.8 (4.8)	-0.0041
Lp(a), mg/dL	777	2.71 (1.21)	316	2.76 (1.17)	316	2.63 (1.22)	-0.0047
C-reactive protein (log), mg/L	775	-1.33 (0.97)	314	-1.41 (0.91)	316	-1.30 (0.98)	-0.0015
Fibrinogen, mg/dL	772	319.9 (76.1)	314	316.8 (71.4)	314	323.5 (77.2)	9.9 E-05
Homocysteine (log), μmol/L	777	2.25 (0.25)	316	2.26 (0.25)	316	2.26 (0.25)	0.0499
Creatinine (log), mg/dL	777	-0.16 (0.26)	316	-0.14 (0.27)	316	-0.13 (0.25)	0.0453
Physical activity	777	-9.89 (7.53)	316	-10.11 (7.71)	316	-9.74 (7.50)	-0.0018
Female, n (%)^c^	777	455 (58.6%)	316	161 (50.9%)	316	163 (51.6%)	-0.0524
Ever smoker, n (%)	777	378 (48.6%)	316	166 (52.5%)	316	151 (47.8%)	-0.0187
Coronary heart disease, n (%)	777	57 (7.3%)	316	25 (7.9%)	316	25 (7.9%)	0.0722
Hypertension, n (%)	777	575 (74.0%)	316	241 (76.3%)	316	240 (75.9%)	-0.0061

^a ^The β estimates and significance values shown are for association with leukoaraiosis volume transformed using the natural logarithm and adjusted for age, sex, and total brain volume.

^b ^The β estimate and significance value shown are for association with leukoaraiosis volume transformed using the natural logarithm and adjusted for sex and total brain volume.

^c ^The β estimate and significance value shown are for association with leukoaraiosis volume transformed using the natural logarithm and adjusted for age and total brain volume.

*p < 0.05; **p < 0.01; ***p < 0.001

BMI, body mass index; SBP, systolic blood pressure; DBP, diastolic blood pressure; HDL, high density lipoprotein; LDL, low density lipoprotein; Lp(a), lipoprotein A

### Associations

Table 2 shows a summary of the results from testing for SNP main effects, SNP-covariate interactions, and SNP-SNP interactions. Of the 1649 SNPs that were evaluated for their association with leukoaraiosis, 37 had FDR<0.3, 15 internally replicated, 23 cross-validated, and only four met all three criteria. In tests for SNP-covariate interaction, 1561 interactions had a FDR<0.3, 834 internally replicated, 1887 cross-validated, and only 12 met all three criteria. In tests for SNP-SNP interactions, one hundred and seventy three SNP-SNP interactions passed all three criteria, and the top 20 most predictive of these interactions are listed in Table 3 along with the single SNP main effects and 12 SNP-covariate interactions that met all three criteria (see Additional file 2 for a complete list of SNP-SNP interactions that passed all three criteria).

**Table 2:** Quantitative summary of genetic associations with leukoaraiosis^a ^that replicated internally, cross-validated, and passed FDR criterion

	**SNP Main Effects**	**SNP-Covariate Interactions**	**SNP-SNP Interactions**
**Number of tests in full sample**	1649	10625	96053
**Model p < 0.10 on full sample**	286	1344	12673
**FDR (q<0.30) on full sample**	37	71	1561
**Replication (Model p < 0.10 in both subsets)**	15	103	834
**Cross-validation (CV R^2 ^> 0.005) on full sample**	23	189	1887
**FDR and Replication**	7	20	281
**FDR and Cross-validation**	22	39	763
**Replication and Cross-validation**	4	30	249
**FDR and Replication and Cross-validation^b^**	4	12	173

^a ^For all associations, the outcome was leukoaraiosis volume transformed using the natural logarithm and adjusted for age, sex, and total brain volume.

^b ^All associations remained significant (p-value < 0.1) in a linear mixed effects model with family as a random effect.

**Table 3:** Genetic effects that replicated internally, cross-validated, and passed FDR criterion

**Main Effects (4)**		**SNP**	**Subset 1 p-value**	**Subset 2 p-value**	**Full Sample p-value**	**R^2 ^× 100**	**CV R^2 ^× 100**
		*F3_rs3917643*	0.0477	0.0270	0.0021	1.58	1.05
		*KITLG_rs995029*	0.0001	0.0921	0.0001	2.33	0.96
		*CAPN10_rs7571442*	0.0856	0.0318	0.0021	1.70	0.59
		*MMP2_rs9928731*	0.0161	0.0383	0.0032	1.48	0.56

**SNP-Covariate Interactions (12)**	**SNP**	**Covariate**	**Subset 1 p-value**	**Subset 2 p-value**	**Full Sample p-value**	**R^2 ^× 100**	**CV R^2 ^× 100**

	*KITLG_rs1492347*	Log homocysteine	0.0002	0.0640	0.0003	4.37	1.57
	*ITGB3_rs3851806*	Height	0.0286	0.0101	0.0010	2.54	1.51
	*TGFB3_rs2284791*	Log C-reactive protein	0.0073	0.0173	0.0003	2.92	1.51
	*TGFB3_rs2284791*	Physical activity	0.0885	0.0015	0.0007	2.69	1.46
	*TGFB3_rs2268622*	Log C-reactive protein	0.0026	0.0360	0.0003	2.86	1.42
	*KITLG_rs995029*	Log homocysteine	0.0002	0.0621	0.0003	4.42	1.40
	*IL28RA_rs11587500*	LDL particle size	0.0026	0.0014	0.0004	2.79	1.26
	*ACCN4_rs1872858*	Fibrinogen	0.0494	0.0339	0.0010	2.59	1.24
	*LTA4H_rs17025079*	Log homocysteine	0.0006	0.0709	0.0001	3.41	1.16
	*PCSK9_rs10888896*	Log homocysteine	0.0239	0.0567	0.0009	2.33	1.13
	*IL22RA1_rs3795299*	CHD	0.0859	0.0138	0.0009	2.41	0.70
	*SERPINE1_rs2227672*	Body mass index	0.0113	0.0219	0.0003	2.69	0.54

**SNP-SNP Interactions (20 of 173)**	**SNP1**	**SNP2**	**Subset 1 p-value**	**Subset 2 p-value**	**Full Sample p-value**	**R^2 ^× 100**	**CV R^2 ^× 100**

	*RHAG_rs11759060*	*GLS_rs1921913*	0.0032	0.0225	8.0 E-06	5.49	2.61
	*F8 _rs7053448*	*MPO_rs34704261*	0.0354	0.0279	0.0001	4.10	2.58
	*RHAG_rs11759060*	*GLS_rs3771316*	0.0032	0.0225	8.0 E-06	5.49	2.55
	*MPO_rs34704261*	*F8_rs1800291*	0.0362	0.0279	0.0001	4.10	2.44
	*SLC20A1_rs10758*	*IL22RA1_rs12093987*	0.0439	0.0085	4.0 E-05	5.44	2.35
	*SLC20A1_rs3827758*	*IL22RA1_rs12093987*	0.0315	0.0915	0.0004	3.88	2.27
	*KITLG_rs995029*	*TLR4_rs1927911*	0.0035	0.0586	5.0 E-05	5.58	2.27
	*SLC20A1_rs1053652*	*IL22RA1_rs12093987*	0.0237	0.0045	5.0 E-05	5.07	2.22
	*MPO_rs34704261*	*F8_rs4898399*	0.0433	0.0315	0.0002	4.18	2.11
	*RHAG_rs2518100*	*GLS_rs1921913*	0.0029	0.0534	0.0001	4.50	2.11
	*MMP2_rs243834*	*IL28RA_rs4330872*	0.0136	0.0856	0.0002	4.46	2.09
	*RHAG_rs2518100*	*GLS_rs3771316*	0.0029	0.0534	0.0001	4.50	1.95
	*MPO_rs8077532*	*F8_rs1800291*	0.0417	0.0802	0.0003	3.93	1.93
	*NMUR1_rs10933376*	*GPR55_rs2969126*	0.0945	0.0022	4.3 E-05	5.03	1.90
	*ACCN4_rs3770234*	*TNFSF10_rs3136596*	0.0246	0.0007	0.0001	4.65	1.84
	*PRKAR2B_rs257376*	*PKRAR2B_rs3729877*	0.0085	0.0027	4.8 E-05	3.55	1.84
	*CX3CR1_rs2853712*	*F2_rs3136435*	0.0153	0.0480	0.0010	3.31	1.82
	*KITLG_rs1492347*	*TLR4_rs10116253*	0.0049	0.0577	0.0001	5.44	1.81
	*F8_rs7053448*	*MPO_rs8077532*	0.0417	0.0802	0.0003	3.93	1.81
	*MPO_rs8077532*	*F8_rs4898399*	0.0515	0.0822	0.0006	4.04	1.81

For all associations, the outcome was leukoaraiosis volume transformed using the natural logarithm and adjusted for age, sex, and total brain volume.

P-value refers to the model p-value (SNP main effects) or the partial F-test p-value of the interaction terms (SNP-Covariate interactions and SNP-SNP interactions).

For SNP-Covariate and SNP-SNP interaction models, CV R^2 ^× 100 refers to the difference in CV R^2 ^× 100 between the full model (including interaction terms) and the reduced model (including main effects only).

*ACCN4 *amiloride-sensitive cation channel neuronal 4;*CAPN10 *calpain 10 (cysteine protease); *CX3CR1 *chemokine, CX3C motif, receptor 1; *F2 *coagulation factor 2; *F3 *coagulation factor 3; *F8 *coagulation factor 8; GLS glutaminase, phosphate activated; *GPR55 *G protein-coupled receptor 55;*IL22RA1 *interleukin 22 receptor, alpha-1;*IL28RA *interleukin 28 receptor; *ITGB3 *integrin, beta-3; *KITLG *KIT tyrosine kinase receptor ligand; *LTA4H *leukotriene A4 hydroxylase; *MMP2 *matrix metalloproteinase 2 (72 kDa type IV collagenase); *MPO *myeloperoxidase; *NMUR1 *neuromedin U receptor 1 (G protein-coupled receptor 66);*PCSK9 *proprotein convertase, subtilisin/kexin-type, 9;*PRKAR2B *protein kinase, cAMP-dependent, regulatory, type II, beta; *RHAG *rhesus blood group-associated glycoprotein; *SERPINE1 *plasminogen activator inhibitor 1; *SLC20A1 *solute carrier family 20 (phosphate transporters), member 1; *TGFB3 *transforming growth factor, beta 3; *TLR4 *toll-like receptor 4; *TNFSF10 *tumor necrosis factor ligand superfamily, member 10

**Figure 2:**
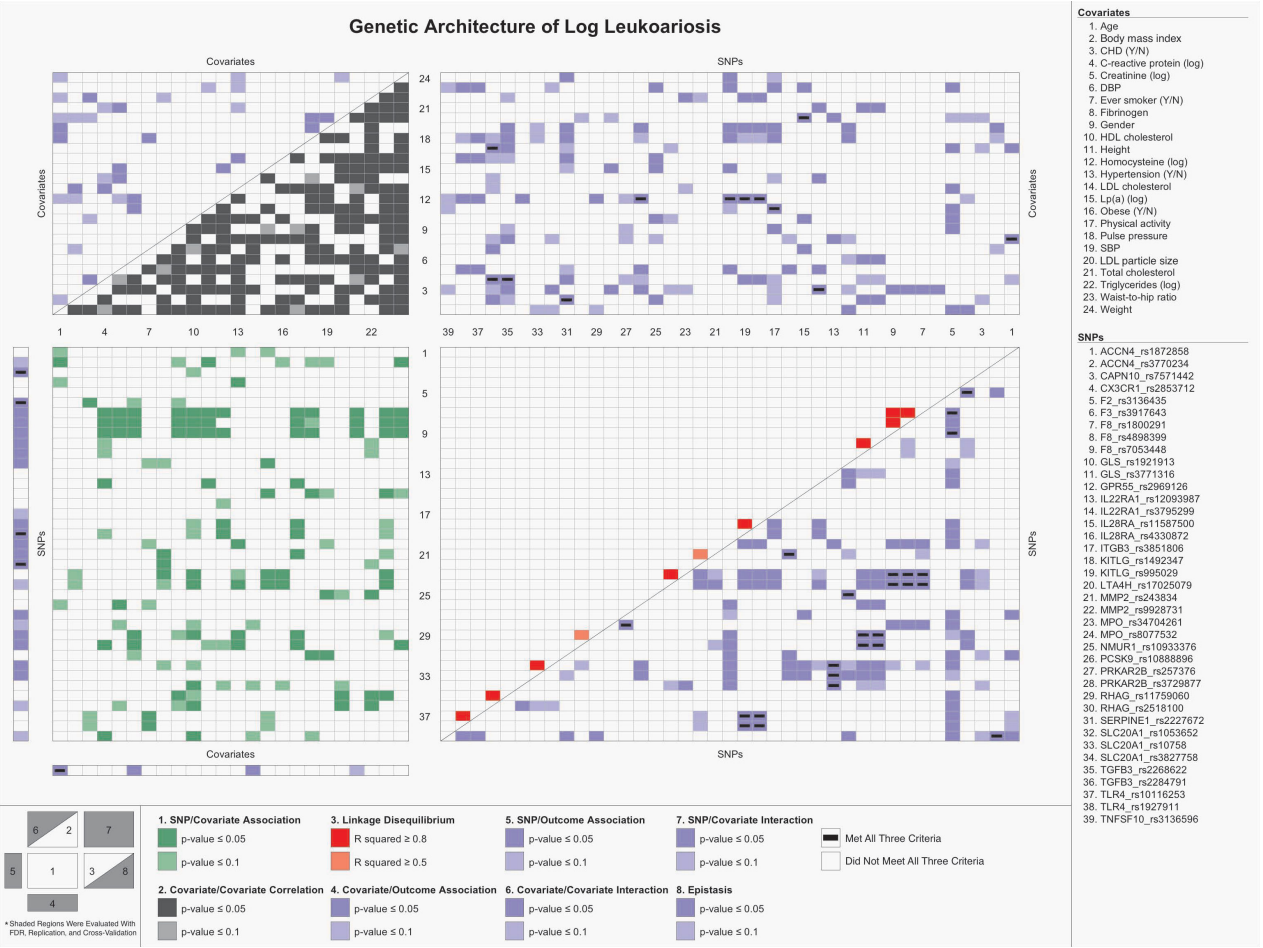
**KGraph: Genetic Architecture of Log Leukoaraiosis**. Using 8 regions, the KGraph shows the relationships between the SNPs, covariates, and outcome by displaying the results from tests of correlation, linkage disequilibrium, association and FDR/Replication/Cross-validation. The key at the bottom of the graphic shows the test criterion for each region and the colors associated with the test result. The region number key in the lower left corner shows the location of each region, and indicates whether the results in the region were assessed using FDR/Replication/Cross-validation (shaded regions). A black bar in the cell indicates that the association passed all three of these criteria. Region 1 displays the association between the SNPs and the covariates, region 2 displays the correlation between the covariates, and region 3 displays the linkage disequilibrium between the SNPs. Region 4 displays covariate association with leukoaraiosis, region 5 displays SNP association with leukoaraiosis, region 6 displays covariate-covariate interactions predicting leukoaraiosis, region 7 displays SNP-covariate interactions predicting leukoaraiosis, and region 8 displays SNP-SNP (epistatic) interactions predicting leukoaraiosis. Included on the KGraph are all of the covariates that were investigated in the study, SNPs that were involved in a single SNP or SNP-covariate association that passed all three filters, and SNPs that were involved in at least one of the 20 most predictive SNP-SNP interactions that passed all three filters.

Figure 2 shows a KGraph, a visual representation of the complex associations among genetic, demographic, and biochemical factors that underlie variation in leukoaraiosis volume. Using both color and spatial relationships, the KGraph presents both associations with leukoaraiosis and the correlation structure of the predictors that underlie those associations. A key to the eight regions of the KGraph is located in the lower left corner of Figure 2. Included on the KGraph are all of the covariates that were investigated in the study, SNPs that were involved in a single SNP or SNP-covariate association that passed all three filters, and SNPs that were involved in at least one of the 20 most highly predictive SNP-SNP interactions that passed all three filters. All associations involving these SNPs and covariates are presented on the KGraph, and those that passed all three filters are indicated by a horizontal black bar.

Region 1 in Figure 2, shown in green, displays the association between the SNPs and covariates, one source of information about the underlying pathways. The majority of SNP-covariate associations were accounted for by three SNPs in the factor VIII (*F8*) gene that were associated with log serum creatinine, height, HDL cholesterol, waist-to-hip ratio, and weight. Region 2, shown in grey, illustrates the correlations between the covariates. The majority of the covariates are significantly associated with one another (p-value < 0.05). Region 3, in red, shows the observed LD, estimated in a sample of 357 unrelated individuals from the study sample. As expected, significant LD (r^2 ^> 0.5) occurs only between SNPs that are within the same gene.

The remaining regions are colored blue, indicating that they represent associations with the outcome of interest, leukoaraiosis. Region 4, which displays the univariate association between the covariates and leukoaraiosis, shows that only age had an association that met all three criteria. Region 5, which illustrates univariate associations between the SNPs and leukoaraiosis, shows that four SNPs have significant, replicated and cross-validated associations (*F3_rs3917643*, *CAPN10_rs7571442*, *MMP2_rs9928731*, *KITLG_rs995029*). Region 6 displays the covariate-covariate interactions that are significantly associated with leukoaraiosis, but no interactions of this type passed all three filters. Region 7 displays the interactions between the SNPs and covariates that were associated with leukoaraiosis. Overall, we detected 12 interactions that replicated and cross-validated, though two pairs of SNP-covariate interactions appear to be marking the same association, due to strong LD between the involved SNPs. Region 8 displays the epistatic (SNP-SNP) interactions significantly associated with leukoaraiosis. We detected 173 replicated and cross-validated, statistically significant pairwise interactions between SNPs. The most predictive interactions included those between SNPs in *RHAG *and *GLS*, *F8 *and *MPO*, *SLC20A1 *and *IL22RA*, *KITLG *and *TLR4*, *NMUR1 *and *GPR55*, *ACCN4 *and *TNFSF10*, and *CX3CR1 *and *F2*. Interactions between two genes that appear more than once in the SNP-SNP results are almost entirely due to strong LD between involved SNPs.

### Predictive Modeling

To begin to assess the combined predictive ability of the top SNPs, covariates, and their interactions, we constructed multiple variable models as described in the Methods section (Table 4). The four single SNPs that met all three criteria explained 5.99% of variation in leukoaraiosis (adjusted R^2^) and had a CV R^2 ^× 100 value of 3.72%. A model that included the main effects and interaction terms from the top four SNP-covariate interactions explained 7.88% of the variation in leukoaraiosis (CV R^2 ^× 100 = 4.53%), while a model including only the SNP and covariate main effect terms had a negative CV R^2^, indicating poor predictive performance. A model consisting of the top four SNP-SNP interactions explained 14.73% of variation in leukoaraiosis (CV R^2 ^× 100 = 9.59%), while the model containing only the SNP main effects explained only 6.12% (CV R^2 ^× 100 = 2.27%), indicating that the SNP-SNP interaction terms explained an additional 7.61% of variation (difference in CV R^2 ^× 100 = 7.32%). Finally, a model that contained both the top four SNP-covariate and the top four SNP-SNP interactions explained 19.18% of the variation in leukoaraiosis (CV R^2 ^× 100 = 11.83%), while the reduced model containing only the SNP and covariate main effects terms explained 7.18% (CV R^2 ^× 100 = 1.30%). Therefore, the combination of SNP-SNP and SNP-covariate interactions was the most predictive model, explaining an additional 12.00% variation in leukoaraiosis (difference in CV R^2 ^× 100 = 10.80%).

**Table 4:** Multivariable analysis to assess combined predictive ability of the best SNPs, covariates, and interactions

**Model**	**Adjusted R^2 ^× 100**	**CV R^2 ^× 100**
1. Top Single SNPs	5.99	3.72

2. Top 4 SNP*Covariate Interactions		
Full Model	7.88	4.53
Reduced Model	2.64	-0.06
Difference^a^		4.53

3. Top 4 SNP*SNP Interactions		
Full Model	14.73	9.59
Reduced Model	6.12	2.27
Difference		7.32

4. Top 4 SNP*Covariate Interactions + Top 4 SNP*SNP Interactions		
Full Model	19.18	11.83
Reduced Model	7.18	1.3
Difference		10.8

5. Single SNPs + Top 4 SNP*Covariate Interactions + Top 4 SNP*SNP Interactions		
Full Model	21.32	11.6
Reduced Model	9.99	2.16
Difference		9.34

^a ^In calculating the difference in CV R^2 ^between full and reduced models, the CV R^2 ^of the reduced model was considered to be zero if it had a negative value.

For all associations, the outcome was leukoaraiosis volume transformed using the natural logarithm and adjusted for age, sex, and total brain volume.

1. Model:*F3_rs3917643 *+*KITLG_rs995029 *+*CAPN10_rs7571442 *+ *MMP2_rs9928731*

2. Full model: KITLG_rs1492347*Log Homocysteine + ITGB3_rs3851806*Height +TGFB3_rs2284791*Log CCRP + TGFB3_rs2284791*Physical Activity; Reduced model:*KITLG_rs1492347 *+ Log Homocysteine + *ITGB3_rs3851806 *+ Height + *TGFB3_rs2284791 *+ Log CCRP + Physical Activity

3. Full model:RHAG_rs11759060*GLS_rs1921913 + F8_rs7053448*MPO_rs34704261 + SLC20A1_rs10758*IL22RA1_rs12093987 + KITLG_rs995029*TLR4_rs1927911; Reduced model: *RHAG_rs11759060 *+ *GLS_rs1921913 *+ *F8_rs7053448 *+ *MPO_rs34704261 *+ *SLC20A1_rs10758 *+ *IL22RA1_rs12093987 *+ *KITLG_rs995029 *+ *TLR4_rs1927911*

4. Full model: KITLG_rs1492347*Log Homocysteine +ITGB3_rs3851806*Height + TGFB3_rs2284791*Log CCRP +TGFB3_rs2284791*Physical Activity + RHAG_rs11759060*GLS_rs1921913 + F8_rs7053448*MPO_rs34704261 + SLC20A1_rs10758*IL22RA1_rs12093987 + KITLG_rs995029*TLR4_rs1927911; Reduced model: *KITLG_rs1492347 *+ Log Homocysteine + *ITGB3_rs3851806 *+ Height + *TGFB3_rs2284791 *+ Log CCRP + Physical Activity + *RHAG_rs11759060 *+ *GLS_rs1921913 *+ *F8_rs7053448 *+ *MPO_rs34704261 *+ *SLC20A1_rs10758 *+ *IL22RA1_rs12093987 *+ *KITLG_rs995029 *+ *TLR4_rs1927911*

5. Full model: *F3_rs3917643 *+*KITLG_rs995029 *+*CAPN10_rs7571442 *+ *MMP2_rs9928731 *+KITLG_rs1492347*Log Homocysteine + ITGB3_rs3851806*Height + TGFB3_rs2284791*Log CCRP + TGFB3_rs2284791*Physical Activity + RHAG_rs11759060*GLS_rs1921913 + F8_rs7053448*MPO_rs34704261 + SLC20A1_rs10758*IL22RA1_rs12093987 + KITLG_rs995029*TLR4_rs1927911; Reduced model: *F3_rs3917643 *+*KITLG_rs995029 *+*CAPN10_rs7571442*+ *MMP2_rs9928731*+*KITLG_rs1492347 + *Log Homocysteine + *ITGB3_rs3851806 *+ Height + *TGFB3_rs2284791 *+ Log CCRP + Physical Activity + *RHAG_rs11759060 *+ *GLS_rs1921913 *+ *F8_rs7053448 *+ *MPO_rs34704261 *+ *SLC20A1_rs10758 *+ *IL22RA1_rs12093987 *+ *TLR4_rs1927911*

## Discussion

Although there have been several studies of the influence of polymorphisms in candidate genes on essential hypertension, stroke, and dementia, little research has been done on the impact of specific candidate gene polymorphisms on leukoaraiosis. Our motivating hypothesis for this work was that polymorphisms in underlying arteriosclerotic pathways may influence leukoaraiosis both directly and through interactions with environmental, demographic, and behavioral risk factors or other genetic polymorphisms.

Except for age and blood pressure, conventional risk factors do not significantly predict leukoaraiosis in our study. However, these covariates predict a large fraction (~30%) of variation in leukoaraiosis. After adjustment for age and sex, four SNPs passed all three filters to reduce false positives and significantly predicted this phenotype. These SNPs represent several distinct physiological pathways, including blood coagulation (*F3*) [31], endothelial and hematopoietic stem cell proliferation (*KITLG*) [32], protease pathways contributing to diabetes (*CAPN10*) [33], and extracellular matrix remodeling (*MMP2*) [34]. This result emphasizes that leukoaraiosis is a complex phenotype that is influenced by genetic variation in several underlying biological processes, in part accounting for inability to predict an individual's leukoaraiosis volume with information regarding conventional and novel risk factors for arteriosclerosis.

In addition to having a significant main effect, the KIT tyrosine kinase receptor ligand (*KITLG*) shows context-dependent effects through interaction with homocysteine and with toll-like receptor 4 (*TLR4*), a mediator of immune response. Several other interactions also suggest a role for immune response and inflammation in the development of leukoaraiosis including gene-environment interactions between *IL28RA *(class II cytokine receptor) and small dense LDL size, *IL22RA1 *(class II cytokine receptor) and coronary heart disease, and both *LTA4H *(leukotriene hydroxylase) and *PCSK9 *(which plays a role in LDL receptor degradation) and homocysteine. Gene-gene interactions that support a role for immunity and inflammation in the disease process include an interaction between *IL22RA1 *and *SLC20A1 *(a receptor for retroviruses) and several interactions between an immune factor and a platelet factor such as that between *MPO *(myeloperoxidase, responsible for microbicidal activity) and platelet factor 8 (*F8*) and between *CX3CR1 *(a cytokine for leukocytes) and platelet factor 2 (*F2*). An interaction between *NMUR1 *(a G-protein coupled activator that appears to be involved in regulation of food intake) and *GPR55 *(a G-protein coupled receptor) also points to genetic variation in signal transduction pathways playing a role in leukoaraiosis development.

Recent work has suggested a number of new potential cellular mechanisms (e.g. endothelial dysfunction, mitochondrial energy metabolism, protein transport) that may play a role in the development of leukoaraiosis and have not been considered previously in candidate gene selection [35,36]. Several unexpected context-dependent effects have also been shown to consistently impact the leukoaraiosis phenotype [37]. In addition, animal and plant studies have recently shown more gene-gene (epistatic) interactions than previously expected [38]. Given the biological complexity of the leukoaraiosis phenotype, it is not surprising that epistatic interactions and context-dependent effects play a large role and explain a larger proportion of variation in the phenotype than single covariate or SNP effects alone in this study. In accordance with this notion, multiple variable predictive modeling that was performed with the most highly ranked single SNP associations, SNP-covariate interactions, and SNP-SNP interactions shows that the variation explained by the SNP-covariate and SNP-SNP interactions (19.18%, CV R^2 ^× 100 = 11.83%) was much higher than that explained by the main effects of these variables alone (7.18%, CV R^2 ^× 100 = 1.30%).

Failure to find replicated SNP effects across studies has significantly limited the utility of genetic association results. Manly [39] suggests that internal validation methods, such as cross-validation, can be implemented as one way to avoid false positives. Cross-validation is an established method for discriminating between true associations and false positives that is based on predictive performance in independent test cases [40], and it has been used in a number of fields that deal with high-dimensional "omics" data [41-44]. Another popular method for reducing false positive associations is to control the false discovery rate, for example, using Storey's q-value [26]. There is a relatively low level of agreement between results filtered through different methods of reducing false positives (FDR q-value < 0.3, internal replication, and cross-validation), emphasizing the need for multiple false positive reduction methods.

Our study has several limitations. The design of the study is based on the premise that susceptibility alleles for common diseases are not under strong selective pressures and are relatively abundant in the population (i.e., the "common disease, common variant" hypothesis). Since the entire allelic spectrum for genes associated with quantitative measures of leukoaraiosis has not been fully delineated, our study was limited to candidate gene choices based on physiological and biological knowledge of leukoaraiosis. In addition, it is possible that multiple rare polymorphisms in the positional and biological candidate genes we studied also influence the phenotype; however, this study was underpowered to detect this type of effect. Since this study was conducted in a cohort of primarily hypertensive non-Hispanic white adults, the inferences may not be generalizable to individuals who are younger, normotensive, or of other ethnicities. Despite these limitations, our approach illustrates the use of polymorphisms in candidate genes to formulate a more complete understanding of the genetic architecture of complex traits such as leukoaraiosis.

## Conclusion

The genetic architecture of complex traits such as leukoaraiosis, a marker of increased risk of stroke and dementia, is comprised of SNP and covariate main effects, gene-gene interactions, and gene-environment interactions from a variety of biological pathways. Our findings indicate that systematic investigation of the context-dependent effects of genetic variation is critical for a more thorough understanding of the multidimensional architecture of complex diseases.

## Abbreviations

The following abbreviations are used in this manuscript: (MRI): Magnetic Resonance Imaging; (GENOA): Genetic Epidemiology Network of Arteriopathy; (GMBI): Genetics of Microangiopathic Brain Injury; (CV): Cross-Validation; (FDR): False Discovery Rate; (SNP): Single Nucleotide Polymorphism; (LD): Linkage Disequilibrium; (MAF): Minor Allele Frequency; (HDL): High Density Lipoprotein; (LDL): Low Density Lipoprotein; (BMI): Body Mass Index; (SBP): Systolic Blood Pressure; (DBP): Diastolic Blood Pressure; (BP): Blood Pressure; (TG): Triglycerides; (FLAIR): Fluid-Attenuated Inversion Recovery; (PCR): Polymerase Chain Reaction.

## Competing interests

The authors declare that they have no competing interests.

## Authors' contributions

SLRK and STT had the original idea for this article, participated in the study design, and participated in discussion of the results. JAS performed the analyses, participated in the study design, participated in discussion of the results, and drafted the manuscript. YVS and MF participated in discussion of the results, RJK assisted with the analysis, THM participated in subject recruitment, IJK measured novel risk factors, and CRJ performed brain imaging. All authors have read and approved the final manuscript.

## Pre-publication history

The pre-publication history for this paper can be accessed here:



## Supplementary Material

Additional File 1 **Summary of Genotyped SNPs in the GENOA-GMBI Study**. This table provides information about the SNPs genotyped in this study, including chromosomal location, nearest gene, genotyping call rate, and Hardy-Weinberg p-value. Click here for file

Additional File 2 **SNP-SNP (Epistatic) Interactions that Passed All Filters**. This table provides information about the 173 epistatic interactions that replicated internally, cross-validated, and passed the FDR criterion. Click here for file
