# Supplementary material for: Complexity in the genetic architecture of leukoaraiosis in hypertensive sibships from the GENOA Study
Source: BMC Med Genomics. 2009 Apr 7;2:16. doi: 10.1186/1755-8794-2-16 (PMC2679055; doi:10.1186/1755-8794-2-16)
Supplement: Additional File 2 — SNP-SNP (Epistatic) Interactions that Passed All Filters. This table provides information about the 173 epistatic interactions that replicated internally, cross-validated, and passed the FDR criterion. [file 1755-8794-2-16-S2.doc]

| **SNP 1** | **SNP 1 Locus** | **SNP 2** | **SNP 2 Locus** | **Subset 1 p-value** | **Subset 2 p-value** | **Full Sample p-value** | **R^2^** | **CV R^2^** | **FDR**  **q-value** |
| --- | --- | --- | --- | --- | --- | --- | --- | --- | --- |
| rs11759060 | RHAG | rs1921913 | GLS | 0.0032 | 0.0225 | 0.0000 | 0.0549 | 0.0261 | 0.0164 |
| rs7053448 | F8 | rs34704261 | MPO | 0.0354 | 0.0279 | 0.0001 | 0.0410 | 0.0258 | 0.0498 |
| rs11759060 | RHAG | rs3771316 | GLS | 0.0032 | 0.0225 | 0.0000 | 0.0549 | 0.0255 | 0.0164 |
| rs34704261 | MPO | rs1800291 | F8 | 0.0362 | 0.0279 | 0.0001 | 0.0410 | 0.0244 | 0.0498 |
| rs10758 | SLC20A1 | rs12093987 | IL22RA1 | 0.0439 | 0.0085 | 0.0000 | 0.0544 | 0.0235 | 0.0330 |
| rs3827758 | SLC20A1 | rs12093987 | IL22RA1 | 0.0315 | 0.0915 | 0.0004 | 0.0388 | 0.0227 | 0.1104 |
| rs995029 | KITLG | rs1927911 | TLR4 | 0.0035 | 0.0586 | 0.0000 | 0.0558 | 0.0227 | 0.0371 |
| rs1053652 | SLC20A1 | rs12093987 | IL22RA1 | 0.0237 | 0.0045 | 0.0000 | 0.0507 | 0.0222 | 0.0184 |
| rs34704261 | MPO | rs4898399 | F8 | 0.0433 | 0.0315 | 0.0002 | 0.0418 | 0.0211 | 0.0757 |
| rs2518100 | RHAG | rs1921913 | GLS | 0.0029 | 0.0534 | 0.0001 | 0.0450 | 0.0211 | 0.0537 |
| rs243834 | MMP2 | rs4330872 | IL28RA | 0.0136 | 0.0856 | 0.0002 | 0.0446 | 0.0209 | 0.0746 |
| rs2518100 | RHAG | rs3771316 | GLS | 0.0029 | 0.0534 | 0.0001 | 0.0450 | 0.0195 | 0.0537 |
| rs8077532 | MPO | rs1800291 | F8 | 0.0417 | 0.0802 | 0.0003 | 0.0393 | 0.0193 | 0.0942 |
| rs10933376 | NMUR1 | rs2969126 | GPR55 | 0.0945 | 0.0022 | 0.0000 | 0.0503 | 0.0190 | 0.0337 |
| rs3770234 | ACCN4 | rs3136596 | TNFSF10 | 0.0246 | 0.0007 | 0.0001 | 0.0465 | 0.0184 | 0.0498 |
| rs257376 | PRKAR2B | rs3729877 | PRKAR2B | 0.0085 | 0.0027 | 0.0000 | 0.0355 | 0.0184 | 0.0359 |
| rs2853712 | CX3CR1 | rs3136435 | F2 | 0.0153 | 0.0480 | 0.0010 | 0.0331 | 0.0182 | 0.1678 |
| rs1492347 | KITLG | rs10116253 | TLR4 | 0.0049 | 0.0577 | 0.0001 | 0.0544 | 0.0181 | 0.0417 |
| rs7053448 | F8 | rs8077532 | MPO | 0.0417 | 0.0802 | 0.0003 | 0.0393 | 0.0181 | 0.0942 |
| rs8077532 | MPO | rs4898399 | F8 | 0.0515 | 0.0822 | 0.0006 | 0.0404 | 0.0181 | 0.1377 |
| rs699 | AGT | rs5987077 | F8 | 0.0165 | 0.0407 | 0.0007 | 0.0385 | 0.0177 | 0.1490 |
| rs4076050 | ADM | rs1805087 | MTR | 0.0837 | 0.0224 | 0.0002 | 0.0350 | 0.0177 | 0.0884 |
| rs3770234 | ACCN4 | rs3136597 | TNFSF10 | 0.0305 | 0.0008 | 0.0001 | 0.0459 | 0.0176 | 0.0643 |
| rs2069824 | IL6 | rs1545970 | ACCN4 | 0.0030 | 0.0050 | 0.0001 | 0.0421 | 0.0175 | 0.0515 |
| rs2069824 | IL6 | rs932307 | SELE | 0.0251 | 0.0337 | 0.0001 | 0.0444 | 0.0174 | 0.0663 |
| rs3093265 | F7 | rs1492347 | KITLG | 0.0096 | 0.0098 | 0.0000 | 0.0588 | 0.0173 | 0.0255 |
| rs3770234 | ACCN4 | rs9859413 | TNFSF10 | 0.0311 | 0.0007 | 0.0001 | 0.0460 | 0.0170 | 0.0646 |
| rs699 | AGT | rs4898398 | F8 | 0.0062 | 0.0665 | 0.0003 | 0.0396 | 0.0167 | 0.0904 |
| rs1006502 | SLC4A5 | rs31563 | IL9 | 0.0460 | 0.0929 | 0.0012 | 0.0374 | 0.0165 | 0.1791 |
| rs3917211 | TGFB3 | rs4927193 | PCSK9 | 0.0324 | 0.0458 | 0.0010 | 0.0327 | 0.0164 | 0.1681 |
| rs3827758 | SLC20A1 | rs877064 | PLA2G2E | 0.0444 | 0.0277 | 0.0007 | 0.0417 | 0.0159 | 0.1449 |
| rs11587500 | IL28RA | rs12460421 | CARM1 | 0.0021 | 0.0486 | 0.0002 | 0.0385 | 0.0159 | 0.0768 |
| rs2069824 | IL6 | rs11835389 | HAL;LTA4H | 0.0223 | 0.0153 | 0.0011 | 0.0247 | 0.0157 | 0.1750 |
| rs17025079 | LTA4H | rs6686007 | IL22RA1 | 0.0439 | 0.0287 | 0.0002 | 0.0396 | 0.0155 | 0.0855 |
| rs7529929 | TNFSF4 | rs9651118 | MTHFR | 0.0448 | 0.0683 | 0.0005 | 0.0283 | 0.0154 | 0.1283 |
| rs3771316 | GLS | rs2180723 | RHAG | 0.0276 | 0.0245 | 0.0003 | 0.0453 | 0.0153 | 0.1011 |
| rs1921913 | GLS | rs2180723 | RHAG | 0.0276 | 0.0245 | 0.0003 | 0.0453 | 0.0153 | 0.1011 |
| rs10779958 | AUP1 | rs2228552 | COL16A1 | 0.0058 | 0.0491 | 0.0000 | 0.0533 | 0.0152 | 0.0191 |
| rs995029 | KITLG | rs10116253 | TLR4 | 0.0036 | 0.0586 | 0.0001 | 0.0554 | 0.0152 | 0.0374 |
| rs909177 | DCTN1 | rs2228552 | COL16A1 | 0.0137 | 0.0128 | 0.0007 | 0.0415 | 0.0149 | 0.1449 |
| rs6048519 | THBD | rs2228552 | COL16A1 | 0.0386 | 0.0046 | 0.0005 | 0.0380 | 0.0148 | 0.1211 |
| rs3770234 | ACCN4 | rs3136602 | TNFSF10 | 0.0311 | 0.0007 | 0.0001 | 0.0460 | 0.0148 | 0.0646 |
| rs2853712 | CX3CR1 | rs3136456 | F2 | 0.0153 | 0.0480 | 0.0010 | 0.0331 | 0.0147 | 0.1678 |
| rs3917211 | TGFB3 | rs11583680 | PCSK9 | 0.0286 | 0.0562 | 0.0013 | 0.0337 | 0.0145 | 0.1857 |
| rs2069824 | IL6 | rs5950587 | AGTR2 | 0.0997 | 0.0244 | 0.0036 | 0.0294 | 0.0145 | 0.2696 |
| rs4149578 | TNFRSF1A | rs1959053 | BDKRB2 | 0.0224 | 0.0119 | 0.0003 | 0.0379 | 0.0145 | 0.0997 |
| rs10758 | SLC20A1 | rs243831 | MMP2 | 0.0091 | 0.0452 | 0.0006 | 0.0436 | 0.0145 | 0.1361 |
| rs7529929 | TNFSF4 | rs2071045 | LEP | 0.0560 | 0.0055 | 0.0002 | 0.0317 | 0.0144 | 0.0884 |
| rs3755065 | ACCN4 | rs3136596 | TNFSF10 | 0.0568 | 0.0201 | 0.0004 | 0.0448 | 0.0143 | 0.1063 |
| rs4236625 | LEP | rs1703081 | KITLG | 0.0000 | 0.0744 | 0.0005 | 0.0326 | 0.0143 | 0.1211 |
| rs1006502 | SLC4A5 | rs932307 | SELE | 0.0731 | 0.0398 | 0.0010 | 0.0427 | 0.0141 | 0.1678 |
| rs17025079 | LTA4H | rs3799675 | RHAG | 0.0592 | 0.0939 | 0.0007 | 0.0438 | 0.0140 | 0.1490 |
| rs995029 | KITLG | rs1155764 | MMP1 | 0.0046 | 0.0799 | 0.0002 | 0.0585 | 0.0138 | 0.0757 |
| rs1545970 | ACCN4 | rs12971616 | CARM1 | 0.0027 | 0.0456 | 0.0012 | 0.0404 | 0.0136 | 0.1793 |
| rs1006502 | SLC4A5 | rs899653 | MARS | 0.0000 | 0.0914 | 0.0000 | 0.0552 | 0.0133 | 0.0200 |
| rs1545970 | ACCN4 | rs12977506 | CARM1 | 0.0028 | 0.0456 | 0.0013 | 0.0403 | 0.0133 | 0.1873 |
| rs2069824 | IL6 | rs8077532 | MPO | 0.0262 | 0.0078 | 0.0005 | 0.0322 | 0.0133 | 0.1310 |
| rs3827758 | SLC20A1 | rs7531863 | PLA2G2E | 0.0508 | 0.0277 | 0.0007 | 0.0416 | 0.0132 | 0.1471 |
| rs13198157 | RHAG | rs1921913 | GLS | 0.0523 | 0.0724 | 0.0017 | 0.0439 | 0.0130 | 0.2037 |
| rs1799964 | LTA;TNF | rs16963927 | CCL5 | 0.0027 | 0.0259 | 0.0028 | 0.0296 | 0.0128 | 0.2444 |
| rs4463047 | FGB | rs3136456 | F2 | 0.0015 | 0.0609 | 0.0010 | 0.0348 | 0.0128 | 0.1680 |
| rs2853550 | IL1B | rs11835389 | LTA4H | 0.0095 | 0.0505 | 0.0008 | 0.0262 | 0.0127 | 0.1600 |
| rs2770381 | AVP | rs11835389 | LTA4H | 0.0470 | 0.0889 | 0.0010 | 0.0294 | 0.0127 | 0.1683 |
| rs1492347 | KITLG | rs1927911 | TLR4 | 0.0048 | 0.0577 | 0.0001 | 0.0548 | 0.0126 | 0.0410 |
| rs3770234 | ACCN4 | rs1959053 | BDKRB2;LOC730199 | 0.0867 | 0.0032 | 0.0023 | 0.0360 | 0.0125 | 0.2238 |
| rs81663 | GP1BA | rs11835389 | HAL;LTA4H | 0.0516 | 0.0042 | 0.0003 | 0.0313 | 0.0124 | 0.0895 |
| rs2275905 | SLC17A4 | rs10012150 | SPP1 | 0.0180 | 0.0559 | 0.0015 | 0.0427 | 0.0124 | 0.1955 |
| rs2239484 | ATP6V1B1 | rs10933376 | NMUR1 | 0.0278 | 0.0337 | 0.0048 | 0.0266 | 0.0123 | 0.2967 |
| rs662 | PON1 | rs243834 | MMP2 | 0.0578 | 0.0245 | 0.0011 | 0.0455 | 0.0123 | 0.1733 |
| rs1799964 | LTA;TNF | rs16971600 | CCL5 | 0.0027 | 0.0259 | 0.0028 | 0.0296 | 0.0122 | 0.2444 |
| rs10758 | SLC20A1 | rs3136594 | TNFSF10 | 0.0055 | 0.0510 | 0.0046 | 0.0399 | 0.0122 | 0.2915 |
| rs699 | AGT | rs5987079 | F8 | 0.0145 | 0.0693 | 0.0015 | 0.0374 | 0.0121 | 0.1934 |
| rs4149579 | TNFRSF1A | rs2069885 | IL9 | 0.0580 | 0.0011 | 0.0001 | 0.0394 | 0.0121 | 0.0498 |
| rs1053652 | SLC20A1 | rs231983 | TNFSF10 | 0.0521 | 0.0220 | 0.0008 | 0.0402 | 0.0120 | 0.1631 |
| rs877064 | PLA2G2E | rs5945128 | F8 | 0.0944 | 0.0109 | 0.0041 | 0.0364 | 0.0120 | 0.2794 |
| rs3755065 | ACCN4 | rs9859413 | TNFSF10 | 0.0574 | 0.0201 | 0.0011 | 0.0426 | 0.0120 | 0.1764 |
| rs17833353 | SLC9A2 | rs6425744 | FABP3 | 0.0984 | 0.0188 | 0.0031 | 0.0370 | 0.0119 | 0.2566 |
| rs4463047 | FGB | rs439401 | APOE;APOC1 | 0.0996 | 0.0063 | 0.0021 | 0.0364 | 0.0119 | 0.2169 |
| rs6919346 | LPA | rs10912560 | TNFSF4 | 0.0667 | 0.0107 | 0.0011 | 0.0300 | 0.0119 | 0.1733 |
| rs3729877 | PRKAR2B | rs12188950 | PART1 | 0.0237 | 0.0930 | 0.0006 | 0.0385 | 0.0119 | 0.1325 |
| rs7149602 | PSMA6 | rs4423707 | CX3CR1 | 0.0933 | 0.0274 | 0.0035 | 0.0234 | 0.0118 | 0.2672 |
| rs9657021 | CYP11B1 | rs3136594 | TNFSF10 | 0.0049 | 0.0810 | 0.0018 | 0.0234 | 0.0118 | 0.2043 |
| rs4149579 | TNFRSF1A | rs17025079 | LTA4H | 0.0312 | 0.0675 | 0.0014 | 0.0350 | 0.0118 | 0.1909 |
| rs1652507 | LPA | rs3136456 | F2 | 0.0206 | 0.0690 | 0.0001 | 0.0392 | 0.0118 | 0.0530 |
| rs2740204 | AVP | rs11835389 | HAL;LTA4H | 0.0431 | 0.0896 | 0.0009 | 0.0307 | 0.0116 | 0.1678 |
| rs740387 | ADD2 | rs2146372 | SERPINC1 | 0.0874 | 0.0319 | 0.0019 | 0.0310 | 0.0116 | 0.2067 |
| rs699 | AGT | rs2096362 | F8 | 0.0145 | 0.0693 | 0.0015 | 0.0374 | 0.0116 | 0.1934 |
| rs1492347 | KITLG | rs1155764 | MMP1 | 0.0043 | 0.0682 | 0.0002 | 0.0579 | 0.0115 | 0.0751 |
| rs1652507 | LPA | rs3136435 | F2 | 0.0206 | 0.0690 | 0.0001 | 0.0392 | 0.0115 | 0.0530 |
| rs1143627 | IL1B | rs12977506 | CARM1 | 0.0519 | 0.0663 | 0.0026 | 0.0381 | 0.0114 | 0.2370 |
| rs822387 | ADIPOQ | rs3861950 | TNFSF4 | 0.0885 | 0.0307 | 0.0025 | 0.0347 | 0.0114 | 0.2320 |
| rs3136598 | TNFSF10 | rs2182833 | PCSK9 | 0.0019 | 0.0737 | 0.0041 | 0.0363 | 0.0114 | 0.2794 |
| rs1800291 | F8 | rs3136435 | F2 | 0.0575 | 0.0225 | 0.0017 | 0.0324 | 0.0113 | 0.2039 |
| rs4463047 | FGB | rs3136435 | F2 | 0.0015 | 0.0609 | 0.0010 | 0.0348 | 0.0110 | 0.1680 |
| rs6076016 | THBD | rs2228552 | COL16A1 | 0.0213 | 0.0331 | 0.0047 | 0.0293 | 0.0109 | 0.2935 |
| rs3770234 | ACCN4 | rs231983 | TNFSF10 | 0.0031 | 0.0843 | 0.0031 | 0.0329 | 0.0109 | 0.2578 |
| rs233998 | TNFSF10 | rs11835389 | LTA4H | 0.0469 | 0.0584 | 0.0028 | 0.0208 | 0.0106 | 0.2451 |
| rs3755065 | ACCN4 | rs3136597 | TNFSF10 | 0.0570 | 0.0202 | 0.0011 | 0.0429 | 0.0105 | 0.1741 |
| rs13198157 | RHAG | rs3771316 | GLS | 0.0523 | 0.0724 | 0.0017 | 0.0439 | 0.0105 | 0.2037 |
| rs740387 | ADD2 | rs16846561 | SERPINC1 | 0.0874 | 0.0405 | 0.0022 | 0.0307 | 0.0103 | 0.2183 |
| rs2268417 | HMGA1L4 | rs3795300 | IL22RA1 | 0.0170 | 0.0276 | 0.0003 | 0.0414 | 0.0103 | 0.1010 |
| rs10755578 | LPA | rs10912560 | TNFSF4 | 0.0393 | 0.0545 | 0.0042 | 0.0311 | 0.0103 | 0.2817 |
| rs2110981 | ADD2 | rs11835389 | LTA4H | 0.0421 | 0.0017 | 0.0035 | 0.0285 | 0.0102 | 0.2661 |
| rs243834 | MMP2 | rs1805087 | MTR | 0.0327 | 0.0958 | 0.0031 | 0.0380 | 0.0102 | 0.2542 |
| rs4236625 | LEP | rs1798011 | KITLG | 0.0000 | 0.0744 | 0.0005 | 0.0326 | 0.0102 | 0.1211 |
| rs2069824 | IL6 | rs34704261 | MPO | 0.0289 | 0.0061 | 0.0013 | 0.0282 | 0.0100 | 0.1865 |
| rs7586970 | TFPI | rs11835389 | LTA4H | 0.0466 | 0.0084 | 0.0021 | 0.0263 | 0.0100 | 0.2169 |
| rs3755065 | ACCN4 | rs12971616 | CARM1 | 0.0059 | 0.0721 | 0.0035 | 0.0437 | 0.0099 | 0.2661 |
| rs5186 | AGTR1 | rs4795095 | CCL5 | 0.0589 | 0.0303 | 0.0018 | 0.0399 | 0.0098 | 0.2046 |
| rs2975766 | CAPN10 | rs697221 | DDIT3 | 0.0436 | 0.0677 | 0.0036 | 0.0347 | 0.0098 | 0.2696 |
| rs1545970 | ACCN4 | rs12093987 | IL22RA1 | 0.0104 | 0.0568 | 0.0030 | 0.0336 | 0.0097 | 0.2535 |
| rs243832 | MMP2 | rs1805087 | MTR | 0.0376 | 0.0870 | 0.0026 | 0.0394 | 0.0097 | 0.2361 |
| rs943580 | AGT | rs9987222 | PLEKHA2 | 0.0054 | 0.0412 | 0.0026 | 0.0327 | 0.0097 | 0.2370 |
| rs1472899 | KITLG | rs1076669 | ECE1 | 0.0085 | 0.0139 | 0.0007 | 0.0353 | 0.0096 | 0.1513 |
| rs7531863 | PLA2G2E | rs5945128 | F8 | 0.0963 | 0.0109 | 0.0041 | 0.0363 | 0.0096 | 0.2794 |
| rs2969126 | GPR55 | rs2192852 | MMP2 | 0.0181 | 0.0834 | 0.0021 | 0.0419 | 0.0096 | 0.2151 |
| rs1053652 | SLC20A1 | rs3136594 | TNFSF10 | 0.0060 | 0.0975 | 0.0031 | 0.0340 | 0.0095 | 0.2542 |
| rs17025079 | LTA4H | rs2180723 | RHAG | 0.0325 | 0.0247 | 0.0014 | 0.0402 | 0.0095 | 0.1913 |
| rs31563 | IL9 | rs11121820 | AGTRAP | 0.0073 | 0.0206 | 0.0003 | 0.0383 | 0.0093 | 0.0942 |
| rs7760223 | RHAG | rs17025079 | LTA4H | 0.0236 | 0.0388 | 0.0008 | 0.0405 | 0.0093 | 0.1592 |
| rs7053448 | F8 | rs3136456 | F2 | 0.0572 | 0.0225 | 0.0017 | 0.0323 | 0.0092 | 0.2039 |
| rs11121819 | AGTRAP | rs31563 | IL9 | 0.0073 | 0.0206 | 0.0003 | 0.0383 | 0.0091 | 0.0942 |
| rs1165165 | SLC17A3 | rs1549926 | CARM1 | 0.0000 | 0.0991 | 0.0000 | 0.0571 | 0.0089 | 0.0147 |
| rs2182833 | PCSK9 | rs6783667 | TNFSF10 | 0.0019 | 0.0737 | 0.0041 | 0.0363 | 0.0088 | 0.2794 |
| rs3136456 | F2 | rs16861205 | ADIPOQ | 0.0005 | 0.0224 | 0.0004 | 0.0328 | 0.0087 | 0.1196 |
| rs4073489 | AGTRAP | rs31563 | IL9 | 0.0089 | 0.0439 | 0.0009 | 0.0347 | 0.0087 | 0.1678 |
| rs4075034 | AGTRAP | rs31563 | IL9 | 0.0071 | 0.0200 | 0.0003 | 0.0385 | 0.0087 | 0.1011 |
| rs3755065 | ACCN4 | rs3136602 | TNFSF10 | 0.0574 | 0.0201 | 0.0011 | 0.0426 | 0.0087 | 0.1764 |
| rs4149579 | TNFRSF1A | rs6838095 | SPP1 | 0.0911 | 0.0808 | 0.0037 | 0.0397 | 0.0086 | 0.2711 |
| rs4842625 | KITLG | rs1076669 | ECE1 | 0.0230 | 0.0137 | 0.0016 | 0.0355 | 0.0085 | 0.1997 |
| rs662 | PON1 | rs11868894 | ITGB3 | 0.0125 | 0.0471 | 0.0019 | 0.0362 | 0.0084 | 0.2088 |
| rs6437000 | HTR2B | rs17025079 | LTA4H | 0.0459 | 0.0268 | 0.0008 | 0.0348 | 0.0084 | 0.1549 |
| rs3755065 | ACCN4 | rs12977506 | CARM1 | 0.0066 | 0.0721 | 0.0039 | 0.0436 | 0.0084 | 0.2771 |
| rs4149579 | TNFRSF1A | rs13144236 | HTRA3 | 0.0617 | 0.0065 | 0.0018 | 0.0317 | 0.0082 | 0.2043 |
| rs1076669 | ECE1 | rs2046971 | KITLG | 0.0085 | 0.0176 | 0.0009 | 0.0353 | 0.0082 | 0.1678 |
| rs3795300 | IL22RA1 | rs13414554 | GLS | 0.0680 | 0.0016 | 0.0035 | 0.0259 | 0.0078 | 0.2688 |
| rs1076669 | ECE1 | rs1000788 | KITLG | 0.0085 | 0.0176 | 0.0010 | 0.0354 | 0.0077 | 0.1678 |
| rs2069824 | IL6 | rs1823227 | TNFSF10 | 0.0868 | 0.0008 | 0.0023 | 0.0297 | 0.0077 | 0.2234 |
| rs13414554 | GLS | rs11868894 | ITGB3 | 0.0812 | 0.0030 | 0.0027 | 0.0311 | 0.0077 | 0.2429 |
| rs13198157 | RHAG | rs17025079 | LTA4H | 0.0496 | 0.0481 | 0.0030 | 0.0403 | 0.0077 | 0.2515 |
| rs10758 | SLC20A1 | rs231983 | TNFSF10 | 0.0452 | 0.0813 | 0.0036 | 0.0405 | 0.0076 | 0.2695 |
| rs3917643 | F3 | rs10487133 | PON2 | 0.0344 | 0.0898 | 0.0041 | 0.0360 | 0.0076 | 0.2794 |
| rs932307 | SELE | rs12087657 | CKS1B | 0.0598 | 0.0161 | 0.0027 | 0.0380 | 0.0075 | 0.2415 |
| rs2302453 | PRKAR2B | rs2228552 | COL16A1 | 0.0823 | 0.0202 | 0.0005 | 0.0395 | 0.0075 | 0.1310 |
| rs2046971 | KITLG | rs31563 | IL9 | 0.0884 | 0.0310 | 0.0034 | 0.0307 | 0.0075 | 0.2634 |
| rs1492347 | KITLG | rs4236625 | LEP | 0.0000 | 0.0351 | 0.0000 | 0.0572 | 0.0075 | 0.0220 |
| rs11888208 | SCN7A | rs171336 | GHRL | 0.0452 | 0.0255 | 0.0015 | 0.0391 | 0.0074 | 0.1946 |
| rs715407 | PRSS25 | rs2227672 | SERPINE1 | 0.0019 | 0.0513 | 0.0000 | 0.0555 | 0.0074 | 0.0147 |
| rs3749172 | GPR35 | rs10211925 | IFNAR2 | 0.0248 | 0.0673 | 0.0043 | 0.0307 | 0.0071 | 0.2845 |
| rs2284791 | TGFB3 | rs5987079 | F8 | 0.0581 | 0.0056 | 0.0039 | 0.0397 | 0.0070 | 0.2771 |
| rs11868894 | ITGB3 | rs3771310 | GLS | 0.0812 | 0.0033 | 0.0029 | 0.0309 | 0.0068 | 0.2485 |
| rs16861205 | ADIPOQ | rs3136435 | F2 | 0.0005 | 0.0224 | 0.0004 | 0.0328 | 0.0067 | 0.1196 |
| rs31563 | IL9 | rs17025079 | LTA4H | 0.0678 | 0.0596 | 0.0027 | 0.0291 | 0.0066 | 0.2407 |
| rs12188950 | PART1 | rs243845 | MMP2 | 0.0990 | 0.0376 | 0.0004 | 0.0418 | 0.0065 | 0.1159 |
| rs10755578 | LPA | rs9500 | S100PBP | 0.0515 | 0.0541 | 0.0018 | 0.0414 | 0.0065 | 0.2043 |
| rs17025023 | LTA4H | rs16861205 | ADIPOQ | 0.0000 | 0.0823 | 0.0000 | 0.0446 | 0.0064 | 0.0156 |
| rs3093265 | F7 | rs995029 | KITLG | 0.0207 | 0.0188 | 0.0001 | 0.0568 | 0.0063 | 0.0503 |
| rs909177 | DCTN1 | rs11835389 | LTA4H | 0.0762 | 0.0776 | 0.0039 | 0.0303 | 0.0063 | 0.2771 |
| rs1006502 | SLC4A5 | rs697221 | DDIT3 | 0.0000 | 0.0815 | 0.0002 | 0.0464 | 0.0063 | 0.0739 |
| rs16861194 | ADIPOQ | rs3136456 | F2 | 0.0021 | 0.0502 | 0.0022 | 0.0267 | 0.0063 | 0.2183 |
| rs3917365 | IL1B | rs11835389 | LTA4H | 0.0104 | 0.0484 | 0.0029 | 0.0217 | 0.0061 | 0.2472 |
| rs995029 | KITLG | rs4236625 | LEP | 0.0000 | 0.0434 | 0.0000 | 0.0571 | 0.0061 | 0.0243 |
| rs2069824 | IL6 | rs10758 | SLC20A1 | 0.0871 | 0.0312 | 0.0048 | 0.0412 | 0.0060 | 0.2967 |
| rs16861194 | ADIPOQ | rs3136435 | F2 | 0.0021 | 0.0502 | 0.0022 | 0.0267 | 0.0059 | 0.2183 |
| rs1143627 | IL1B | rs12971616 | CARM1 | 0.0444 | 0.0663 | 0.0023 | 0.0382 | 0.0057 | 0.2255 |
| rs877064 | PLA2G2E | rs1805087 | MTR | 0.0087 | 0.0506 | 0.0013 | 0.0359 | 0.0055 | 0.1901 |
| rs2072246 | ADD2 | rs4220_x | NA | 0.0411 | 0.0989 | 0.0032 | 0.0376 | 0.0054 | 0.2602 |
| rs2969126 | GPR55 | rs10751768 | IL22RA1 | 0.0759 | 0.0684 | 0.0019 | 0.0348 | 0.0054 | 0.2067 |
| rs1992188 | GPR55 | rs1892251 | SLC17A4 | 0.0036 | 0.0701 | 0.0045 | 0.0448 | 0.0052 | 0.2897 |
| rs2969126 | GPR55 | rs2146372 | SERPINC1 | 0.0798 | 0.0044 | 0.0013 | 0.0379 | 0.0052 | 0.1861 |
| rs1801253 | ADRB1 | rs7690786 | HTRA3 | 0.0973 | 0.0694 | 0.0023 | 0.0332 | 0.0052 | 0.2234 |
| rs7053448 | F8 | rs3136435 | F2 | 0.0572 | 0.0225 | 0.0017 | 0.0323 | 0.0051 | 0.2039 |
| rs31563 | IL9 | rs1000788 | KITLG | 0.0891 | 0.0310 | 0.0034 | 0.0307 | 0.0051 | 0.2653 |
